# Transient loss of venous integrity during developmental vascular remodeling leads to red blood cell extravasation and clearance by lymphatic vessels

**DOI:** 10.1242/dev.156745

**Published:** 2018-02-01

**Authors:** Yang Zhang, Nina Daubel, Simon Stritt, Taija Mäkinen

**Affiliations:** Uppsala University, Department of Immunology, Genetics and Pathology, Dag Hammarskjölds väg 20, 751 85 Uppsala, Sweden

**Keywords:** Platelet, Lymphvasculogenesis, Endothelial integrity, Blood-filled lymphatic vessel

## Abstract

Maintenance of blood vessel integrity is crucial for vascular homeostasis and is mainly controlled at the level of endothelial cell (EC) junctions. Regulation of endothelial integrity has largely been investigated in the mature quiescent vasculature. Less is known about how integrity is maintained during vascular growth and remodeling involving extensive junctional reorganization. Here, we show that embryonic mesenteric blood vascular remodeling is associated with a transient loss of venous integrity and concomitant extravasation of red blood cells (RBCs), followed by their clearance by the developing lymphatic vessels. In wild-type mouse embryos, we observed activated platelets extending filopodia at sites of inter-EC gaps. In contrast, embryos lacking the activatory C-type lectin domain family 1, member b (CLEC1B) showed extravascular platelets and an excessive number of RBCs associated with and engulfed by the first lymphatic EC clusters that subsequently form lumenized blood-filled vessels connecting to the lymphatic system. These results uncover novel functions of platelets in maintaining venous integrity and lymphatic vessels in clearing extravascular RBCs during developmental remodeling of the mesenteric vasculature. They further provide insight into how vascular abnormalities characterized by blood-filled lymphatic vessels arise.

## Abstract (precis)

**Highlighted Article:** Previously unrecognized roles of platelets and lymphatic vessels during developmental vascular remodeling are revealed, providing mechanistic insight into how vascular abnormalities characterized by blood-filled lymphatic vessels arise.

## INTRODUCTION

Maintenance of the integrity of blood vessels is crucial for vascular homeostasis, and its disruption can lead to hemorrhage, edema, inflammation and tissue ischemia (Murakami and Simons, 2009). Endothelial integrity and barrier properties are mainly controlled at the level of endothelial cell-cell junctions. Associated mural cells and the basement membrane (BM) stabilize the vessel wall and form an additional barrier that prevents leakage. Disruption of cell-cell junctions can be accompanied by exposure of the thrombogenic extracellular matrix of the vessel wall, which triggers thrombus formation. Thereby, platelets also play an important role in vascular homeostasis by sealing gaps in the injured endothelium. They can additionally promote barrier function in resting endothelium by releasing a variety of soluble factors (Ho-Tin-Noé et al., 2011).

Most studies have focused on the role of platelets in the maintenance of the mature vasculature, but their role in promoting neo-angiogenesis and maintaining integrity of angiogenic vessels in adult tissues has also been reported (Ho-Tin-Noé et al., 2011). Platelets are thought to be dispensable for embryonic vessel integrity with the exception of the cerebral vasculature, in which interaction of platelet CLEC1B (also known as CLEC2) with podoplanin (PDPN) on the neuroepithelium is required for platelet adhesion, aggregation and secretion (Lowe et al., 2015). Although CLEC1B appears to have a minimal role in physiological hemostasis (Bender et al., 2013), it is important for platelet function in preventing inflammation-induced hemorrhaging (Boulaftali et al., 2013) and mediating initiation of deep vein thrombosis (Payne et al., 2017). In addition, platelet dysfunction caused by *Clec1b* deficiency in mice has been associated with abnormal blood filling of lymphatic vessels (Welsh et al., 2016). However, this phenotype was shown to be caused by back-filling of lymphatic vessels with blood due to defective platelet aggregation and thrombus formation at the lymphovenous junction (Hess et al., 2014), rather than defects in the blood vasculature.

Here, we studied the regulation of vessel integrity during embryonic vascular morphogenesis. We found that remodeling of the mesenteric blood vasculature is associated with a transient disruption of venous endothelial integrity. We further identify previously unrecognized roles of platelets and lymphatic vessels during developmental vessel remodeling in maintaining endothelial integrity and clearing of red blood cells (RBCs), respectively.

## RESULTS AND DISCUSSION

### Transient extravasation of RBCs and their engulfment by lymphatic vessels during mesenteric vascular development

**Fig. 1.**
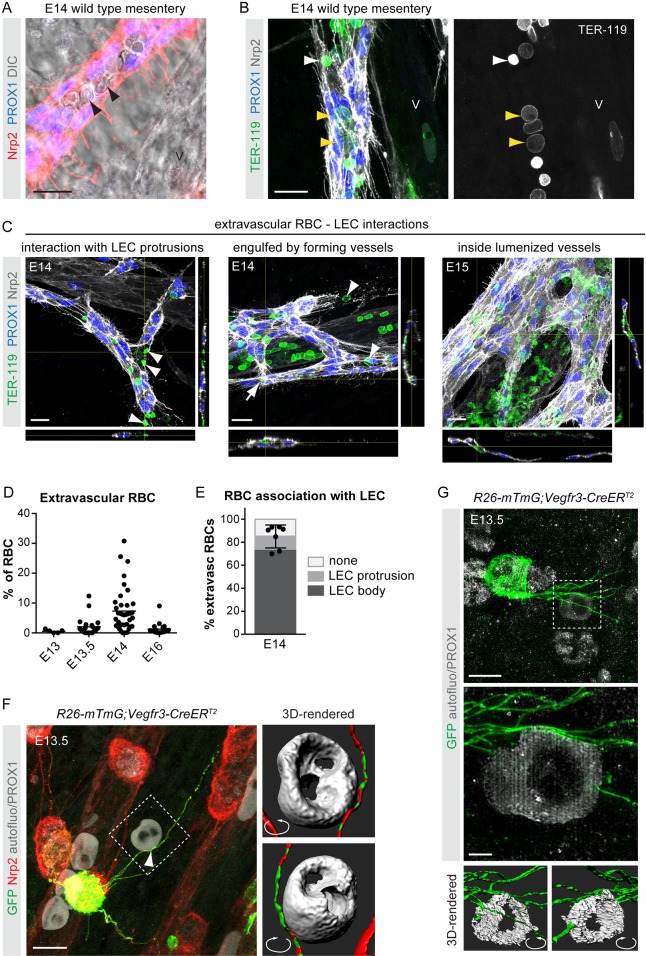
**Transient RBC extravasation during the development of the mesenteric vasculature.** (A,B) Whole-mount immunofluorescence (A,B) and DIC imaging (A) of E14 mesenteries showing RBCs inside lymphatic vessels (arrowheads). Both nucleated (TER-119^low^, yellow arrowheads in B) and enucleated (TER-119^high^, white arrowhead in B) RBCs were present. V, mesenteric vein. (C) Whole-mount immunofluorescence of mesenteric vessels for the indicated antibodies showing TER-119^+^ RBC interactions with Nrp2^high^ LEC protrusions (E14; on the left), and engulfment by forming lymphatic vessels (E14; in the middle) and lumenized lymphatic vessels (E15; on the right). *z*-views at the indicated positions are shown below and on the right of each image. (D) The percentage of extravascular RBCs versus total (intravascular and extravascular) RBCs in the mesentery at the indicated stages of development. Dots represent individual embryos (E13: *n*=5) or vessels (E13.5: *n*=23; E14: *n*=39; E16: *n*=37) and horizontal lines represent mean values (see Materials and Methods for details). (E) The percentage of extravascular RBCs at E14 showing association with LEC protrusions (light gray), the LEC cell body (in most cases engulfment of RBCs by a cluster of LECs, and less frequently localization next to a cluster but associated with the cell body of an LEC; dark gray), or no association with LECs (white). Dots represent individual embryos (*n*=7), mean±s.d. (F) Deconvolved confocal image showing contact (arrowhead) between a protrusion from a mesenteric LEC (visualized by Nrp2 and PROX1 staining and GFP expression in a mosaically induced *R26-mTmG;Vegfr3-CreER^T2^* embryo) and extravascular RBC (visualized by autofluorescence signal). Boxed area is shown on the right as a surface-rendered 3D image from angles that reveal association between RBC and LEC protrusion. (G) Reconstructed SIM images showing direct contacts between LEC (PROX1^+^GFP^+^) protrusions and a RBC (autofluorescence signal) in E13.5 *R26-mTmG;Vegfr3-CreER^T2^* mesentery. Boxed area is shown below as a digital zoom-in of maximum intensity projection of central part of the image stack and as surface-rendered 3D reconstructions of whole image stack viewed from opposite angles illustrating how LEC protrusions align with the surface of the RBC. Scale bars: 20 μm (A-C); 10 μm (F,G, upper panel); 2 μm (G, lower panel).

Most lymphatic vessels in mammals have been described to form through lymphangiogenic sprouting from embryonic veins (Srinivasan et al., 2007). In contrast, lymphatic vessels in the mesentery form through lymphvasculogenic assembly of lymphatic endothelial cell (LEC) progenitors into clusters that further coalescence to lumenized vessels between embryonic day (E) 13 and E14 (Stanczuk et al., 2015). Immunofluorescence combined with differential interference contrast (DIC) imaging of E14 mesenteries from wild-type embryos unexpectedly showed the presence of cells characteristic of RBCs inside the developing lymphatic vessels (Fig. 1A). TER-119 (LY76) staining confirmed RBC identity and revealed both nucleated (TER-119^low^) and enucleated (TER-119^high^) RBCs inside lymphatic vessels (Fig. 1B).

To investigate the significance of this phenomenon, we first determined the frequency of extravascular RBCs in the developing mesentery based on whole-mount immunofluorescence for markers of RBCs as well as blood and lymphatic endothelial cells (ECs) (Fig. 1C, Movie 1, Fig. S1A). Rare extravascular RBCs were observed at E13-E13.5 [0.55±0.57% (*n*=5) and 2.05±3.07% (*n*=23) of total RBCs, respectively; Fig. 1D], but the frequency increased to 7.38±7.33% (*n*=39) at E14 (Fig. 1C,D). Notably, the majority of extravascular RBCs interacted with or were captured within LEC membrane protrusions, or engulfed by LEC clusters at E14 (Fig. 1C,E, Movie 1). At E15, RBCs were frequently observed in the lumen of the developing lymphatic vessels (Fig. 1C), but were no longer present after E16 [Fig. 1D; 1.26±2.00% extravascular RBCs (*n*=37)], once lymphatic drainage is initiated (Sabine et al., 2012).

To improve our understanding of the mechanisms leading to RBC interaction with and engulfment by LECs, we analyzed mesenteries at E13-E13.5 when LEC clusters first appear. Consistent with the low number of extravascular RBCs, most LEC clusters did not interact with RBCs at this stage (Fig. S1B), suggesting that LEC emergence and cluster formation is not controlled by extravascular RBCs. To investigate LEC-RBC interactions at high resolution, we labeled individual LECs mosaically by Cre-activated expression of a membrane-bound GFP in *R26-mTmG;Vegfr3-CreER^T2^* embryos (Martinez-Corral et al., 2016) using a suboptimal dose of 4-hydroxytamoxifen. Autofluorescence signal in combination with PROX1 staining allowed visualization of RBCs and LEC nuclei, respectively. Deconvolved confocal images showed close association of RBCs with mesenteric LECs at E13.5 (Fig. 1F, Movie 2). Imaging of whole-mount mesenteries with structured illumination microscopy (SIM) and 3D reconstruction confirmed direct contacts between LEC protrusions and RBCs (Fig. 1G, Movie 3). Despite the observed interaction of RBCs with LEC clusters at E14 (Fig. 1E), LECs were frequently found to extend protrusions initially in a random manner (Fig. S1C), arguing against a specific RBC-derived chemoattractant in driving the formation of LEC protrusions.

Taken together, these results indicate transient RBC extravasation during the development of the mesenteric vasculature. Selective association of RBCs with LECs further suggests a role for developing lymphatic vessels in the capture and clearance of extravasated RBCs.

### Remodeling of the developing mesenteric blood vasculature is associated with a transient loss of endothelial integrity

**Fig. 2.**
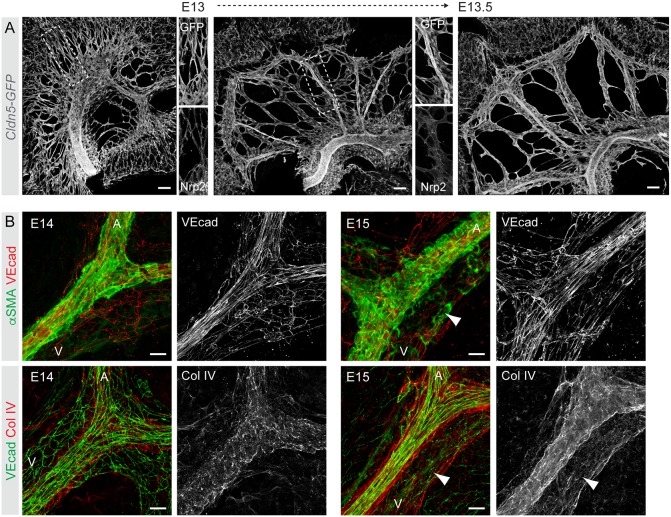
**Mesenteric blood vasculature undergoes extensive remodeling and maturation between E13** **and** **E15.** (A) Visualization of the mesenteric vasculature in *Cldn5-GFP* embryos showing extensive remodeling from a primary plexus into a segmentally organized pattern of veins and arteries between E13 and E14. Single-channel images of the boxed areas show co-staining for the venous EC marker Nrp2 in only a subset of vessels. (B) Whole-mount immunofluorescence of E14 (left) and E15 (right) mesenteric vessels for markers of ECs (VE-cad; Cdh5), mural cells (αSMA; Acta2) and basement membrane (collagen IV). Single-channel images of indicated stainings are shown. Note poor EC alignment as well as mural cell and BM coverage in E14 vein (V) compared with E15 vein (arrowheads), or the artery (A). Scale bars: 100 μm (A); 20 μm (B).

In the tumor vasculature, extravasation of RBCs leading to hemorrhage is associated with disruption of EC integrity (Hashizume et al., 2000). To analyze the integrity of the developing mesenteric blood vessels, we first visualized the morphological changes in the vasculature at the critical stages of development using a *Cldn5-GFP* reporter, which allows visualization of all ECs by strong GFP fluorescence (Stanczuk et al., 2015). As previously reported (Hatch and Mukouyama, 2015), at E13 mesenteric blood vessels form a primary plexus that remodels by E13.5 into a segmentally organized pattern of veins and arteries running in parallel (Fig. 2A). Arterial-venous identity was established prior to remodeling, as indicated by expression of the venous EC marker Nrp2 in only a subset of vessels within the primitive plexus (Fig. 2A). At E14, arteries had a continuous BM and extensive mural cell coverage (Fig. 2B), typical of mature vessels. In contrast, E14 mesenteric veins had only a fragmented BM and few mural cells (Fig. 2B).

**Fig. 3.**
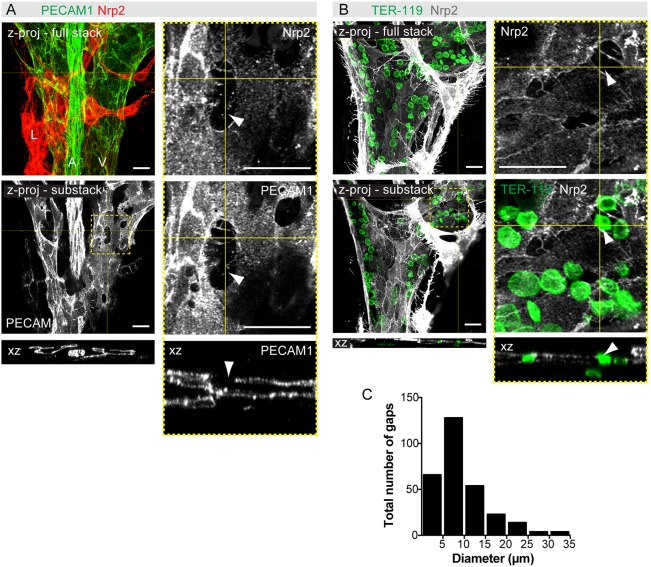
**Remodeling of the mesenteric blood vasculature is associated with a transient loss of venous endothelial integrity.** (A,B) Whole-mount staining of E14 mesenteries for the indicated antibodies, showing intercellular gaps in the veins. *z*-projections of confocal stacks are shown and boxed areas are magnified on the right. *z*-views at the indicated positions are shown below. Arrowheads indicate gaps in the endothelial layer. (C) Size distribution of intercellular gaps in wild-type E14 mesenteric veins (*n*=294 gaps from 14 embryos). Scale bars: 20 μm.

ECs of E14 arteries were aligned along the longitudinal axis of the vessels in the direction of flow and showed continuous cell-cell junctions (Fig. 2B; data not shown). In contrast, E14 mesenteric veins unexpectedly showed disrupted cell-cell junctions between adjacent venous ECs, characterized by large intercellular gaps and filopodial extensions (Fig. 3A,B, Movies 4, 5). Intercellular gaps were observed by staining with both Nrp2 and PECAM1 antibodies (Fig. 3A). Co-staining with TER-119 antibodies showed the presence of RBCs in the intercellular gaps at the level of the EC layer and protrusion into the extravascular space (Fig. 3B). Most endothelial gaps were 6-10 µm in diameter (Fig. 3C), with an average diameter of 9.5±6.2 μm (*n*=294), and thus permissive for extravasation of both enucleated (6 μm diameter) and nucleated (8 μm diameter) RBCs. Concomitant with increased mural cell recruitment and BM deposition (Fig. 2B), intercellular gaps were no longer detected in E15 mesenteric veins (data not shown). These observations suggest that normal vascular remodeling in the mesentery involves a transient loss of venous endothelial integrity, which correlates with extravasation of RBCs.

### Platelets maintain venous integrity and prevent excessive RBC extravasation during mesenteric vascular remodeling

**Fig. 4.**
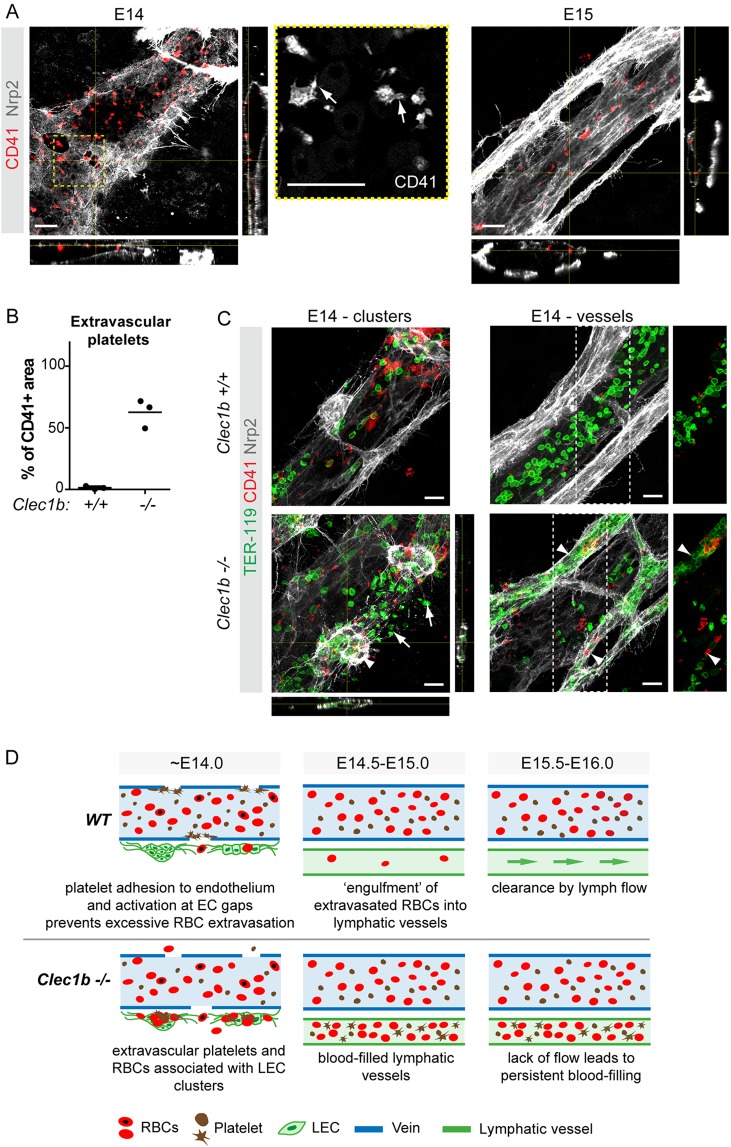
**Platelets are crucial for maintaining integrity of remodeling veins.** (A) Adherence of platelets (CD41^+^) to venous ECs (Nrp2^low^) and gaps at E14, but not at E15. *z*-views of the stack at the indicated positions are shown and boxed area is magnified on the right. Note filopodia extension (arrows in the magnified image showing CD41 staining alone), indicative of platelet activation, at E14. (B) Quantification of extravascular (LEC-associated) platelets in E14 wild-type and *Clec1b^−/−^* mesenteries. Dots represent individual embryos (*n*=3, mean of three areas imaged for each embryo) and the horizontal lines represent mean values. (C) Whole-mount immunofluorescence of E14 wild-type and *Clec1b^−/−^* mesenteries for TER-119 (RBCs), CD41 (platelets) and Nrp2 [lymphatic vessels (high) and veins (low)]. Note the presence of RBCs and platelets in association with LEC clusters (E14 - clusters panel, arrowhead) and in the extravascular space (arrows), as well as in lumenized lymphatic vessels (E14 - vessels panel, arrowheads) in *Clec1b^−/−^* but not wild-type mesenteries. RBC and platelet staining of the boxed areas are shown to the right (E14 - vessels panels). *z*-views of the stack at the indicated positions are shown below and on the right of the bottom panel E14 - clusters. (D) Proposed model of platelet function in mesenteric vascular development and the mechanism underlying blood-filling of lymphatic vessels in *Clec1b^−/−^* embryos. Scale bars: 20 μm.

Platelets form aggregates to limit blood loss and plasma leakage in primary homeostasis and upon vascular injury (Ho-Tin-Noé et al., 2011). We hypothesized that they are also involved in maintaining vessel integrity during mesenteric vascular remodeling. Staining for the marker CD41 (ITGA2B) revealed the presence of platelets at endothelial gaps, but also at the endothelium in areas where no gaps were detected (Fig. 4A). Although large aggregates were not observed, filopodia extension was indicative of platelet activation (Fig. 4A). Notably, unlike RBCs, platelets were found neither outside of the blood vessels, nor in association with LECs (Fig. 4A,B), suggesting that their activation prevents extravasation. Lack of endothelial association at E15 further suggests that platelets adhere to the endothelial layer only at the stage when intercellular gaps are present (Fig. 4A).

To investigate the functional importance of platelets in the maintenance of venous integrity, we used *Clec1b*-deficient mice (*Clec1b^−/−^*), which show impaired platelet function leading to decreased thrombus stability (May et al., 2009; Suzuki-Inoue et al., 2010), and, intriguingly, blood-filled lymphatic vessels (Bertozzi et al., 2010; Finney et al., 2012; Suzuki-Inoue et al., 2010). The latter phenotype was initially described as a failure in the developmental separation of the two vascular systems following the formation of the first lymphatic vessels via sprouting from major veins. More recently, the underlying mechanism was assigned to back-filling of lymphatic vessels with blood from the thoracic duct due to defective platelet aggregation and thrombus formation at the lymphovenous junction (Hess et al., 2014; Welsh et al., 2016). In agreement with previous studies, we found that mesenteric lymphatic vessels of E14 *Clec1b*^−/−^ embryos were filled with RBCs (Fig. 4C). Notably, *Clec1b*^−/−^ mesenteries also showed extravascular platelets that were associated with LECs (Fig. 4B,C). Both platelets and RBCs were found in the interstitial space and associated with LEC clusters at E14 but prior to establishment of lumenized lymphatic vessels in the mesentery (Fig. 4C) and skin (Fig. S2) of *Clec1b*^−/−^ mutants. The observation that *Clec1b^−/−^* embryos show extravascular RBCs and platelets prior to establishment of functional lumenized lymphatic vessels that are connected to the circulation argues that their filling with blood occurs secondary to disruption of blood vessel integrity due to platelet dysfunction.

These findings raise a question on the mechanism of CLEC1B-dependent activation of platelets at venous endothelial gaps. Podoplanin is so far the only known endogenous ligand of CLEC1B (Navarro-Núñez et al., 2015; Pollitt et al., 2014), and *Pdpn* knockout mice also show blood-filling of lymphatic vessels (Fu et al., 2008; Uhrin et al., 2010). Podoplanin is highly expressed in mesenteric LEC clusters at E14 (Fig. S3A,B; Stanczuk et al., 2015), but also in non-ECs (Fig. S3A,B). This is in agreement with previous reports showing expression of podoplanin in multiple cell types, of which mesothelial cells (Schacht et al., 2005), leukocytes (Kerrigan et al., 2012; Lee et al., 2010) and stromal fibroblasts (Kawase et al., 2008; Christer Betsholtz, personal communication) might be relevant in the context of the developing mesentery. To characterize the PDPN^+^ cells further, we utilized embryos carrying the *Pdgfrb-eGFP* transgene, which labels mural cells (He et al., 2016) and a large stromal cell population in E14 mesenteries (Fig. S3B). Fluorescence-activated cell sorting (FACS) analysis of E14 mesenteries showed three distinct podoplanin-expressing cell populations: PECAM1^+^PDPN^high^GFP^−^ LECs; PECAM1^−^ stromal cells, of which PDPN^intermed^GFP^−^ cells likely represent mesothelial cells; and PDPN^low^GFP^+^ fibroblasts (Fig. S3C). Thus, at least three populations of PDPN-expressing cells (LECs, mesothelial cells and fibroblasts) are in the immediate vicinity of the developing mesenteric veins and might be involved in establishing endothelial integrity through interaction with platelet CLEC1B. Generation of mice lacking podoplanin in specific cell types during embryonic development will be important for addressing this question in the future. Notably, postnatal LEC-specific deletion of *Pdpn* leads to progressive blood-filling of lymphatic vessels over a few weeks (Bianchi et al., 2017). The mechanism is, however, different from the embryonic process and is attributed in both *Pdpn*- and *Clec1b*-deficient mice to back-filling of the thoracic duct and lymph nodes with blood through the lymphovenous connection (Bianchi et al., 2017; Hess et al., 2014). Yet another CLEC1B-PDPN-mediated mechanism safeguarding the lymphatic vasculature has been described in the lymph node, where the integrity of high endothelial venules (HEVs) was shown to depend on the interaction between PDPN in fibroblastic reticular cells, surrounding HEVs, and platelet CLEC1B (Herzog et al., 2013). Similarly, interaction of platelet CLEC1B with PDPN on the neuroepithelium is required for the maintenance of cerebrovascular integrity (Lowe et al., 2015).

Taken together, our study demonstrates that embryonic remodeling of the mesenteric blood vasculature is associated with a transient loss of venous integrity due to formation of large intercellular gaps in the endothelium and consequent RBC extravasation. Such a disruption of vascular integrity has been observed in pathological conditions (Hashizume et al., 2000; Mazzone et al., 2009), but our results show that it also occurs as part of a normal developmental process. We further show that platelets are essential for the maintenance of venous integrity and prevent excessive RBC extravasation during mesenteric vascular remodeling, while lymphatic vessels clear the extravasated RBCs from the tissue (Fig. 4D). It is appealing to speculate that CLEC1B represents an embryonic mechanism of platelet activation when BM collagens, which provide a major platelet activation signal in the mature vasculature (Dütting et al., 2012), are scarce. Finally, our data provide mechanistic insight into how vascular abnormality characterized by blood-filled lymphatic vessels arises, by showing that it can occur secondary to loss of venous integrity. Notably, the blood-filled lymphatic vessel phenotype has been observed in various genetic models and has been considered as direct evidence of primary lymphatic vascular defects. In light of our findings, defects in the blood vasculature should also be considered.

## MATERIALS AND METHODS

### Mice

*Vegfr3-CreER^T2^* (Martinez-Corral et al., 2016), *R26-mTmG* (Muzumdar et al., 2007), *Cldn5-GFP* (a gift from C. Betsholtz, Uppsala University, Sweden), *Clec1b^flox^* (Acton et al., 2014), *Pdgfrb-eGFP* (He et al., 2016) and wild-type mice were analyzed on a C57BL/6J background. *Clec1b^flox^* were crossed with *PGK-Cre* mice to generate germline heterozygous mice that were further crossed to generate germline homozygous embryos. The morning of vaginal plug detection was considered as embryonic day 0. Mosaic labeling of individual LECs by membrane-bound GFP in E13 *R26-mTmG;Vegfr3-CreER^T2^* embryos was induced by administration of 0.5 mg of 4-hydroxytamoxifen (Sigma-Aldrich) to pregnant females at E12. Experimental procedures were approved by the Uppsala Laboratory Animal Ethical Committee.

### Whole-mount immunofluorescence

Mesenteries were fixed in 4% paraformaldehyde at room temperature for 2 h and stained as previously described (Stanczuk et al., 2015). The following primary antibodies were used: mouse anti-α-smooth muscle actin-Cy3 (Sigma-Aldrich, C6198, 1:250), rat anti-endomucin (V.7C7) (Santa Cruz, SC-65495, 1:200), rat anti-CD41-FITC (eBioscience, 11-0411-81, 1:50), rabbit anti-collagen IV (Bio-Rad, 2150-1470, 1:500), chicken anti-GFP (Abcam, ab13970, 1:200), goat anti-neuropilin 2 (R&D Systems, AF567, 1:200), hamster anti-PECAM1 (Millipore, MAB1398Z, 1:1000), rat anti-PECAM1 (BD Pharmingen, 553370, 1:1000), rat anti-PECAM1-AF594 (BioLegend, 102520, 1:100), rabbit anti-human PROX1 (Stanczuk et al., 2015; 1:200), rat anti-TER-119 (eBioscience, 145921, 1:200), rat anti-TER-119-AF647 (BioLegend, 116218, 1:50), goat anti-VE-cadherin (Santa Cruz, SC-6458, 1:200) and anti-podoplanin (Developmental Studies Hybridoma Bank at the University of Iowa, clone 8.1.1, 1:800). Autofluorescence signal at 550-600 nm wavelength was used for visualization of RBCs. Secondary antibodies conjugated to AF488, AF594, AF647 or Cy3 were from Jackson ImmunoResearch, and all were used at 1:300.

### Image acquisition and quantification

Confocal image stacks were acquired using a Leica SP8 confocal microscope and LAS X software. Images represent maximum intensity projections of tiled *z*-stacks. Images were processed with Fiji or Adobe Photoshop software. Deconvolution (Fig. 1F) was carried out in Huygens Essential v16.5 (Scientific Volume Imaging) using a theoretical point spread function and automatic background estimation. Stopping criteria were set to 40 iterations and a signal-to-noise ratio of 10. Structural illumination raw image stacks were acquired on a Zeiss Elyra S.1 LSM710 together with a Plan-Apochromat 63×/1.4 Oil DIC M27 objective. GFP and Cy3 were imaged sequentially as separate tracks and excited at 488 and 561 nm, respectively, using the same grid size of 28 µm. For detection, beam splitters BP 495-550+LP 750 and BP 570-620+LP 750 were used. Tracks were switched for each *z*-stack. Reconstruction of structural illumination raw images was carried out using the SIM module of ZEN software (Carl Zeiss). The noise filter was manually set to −4.0. Same parameters were set for both channels. Maximum intensity projections of deconvolved or reconstructed image stacks were generated using Fiji. Movies 1, 4 and 5 were generated in Fiji and recorded at 3 frames per second (fps). Movies 2 and 3 were generated in Imaris v8.4 (Bitplane) and recorded at 25 fps. Image acquisition details are provided in Table S1.

For quantification of intra/extravascular RBCs, mesenteries were stained for TER-119, PROX1 and Nrp2 (all samples), and PECAM1 (E13, E13.5 and E14 samples) to label RBCs, LECs and (venous) blood ECs, respectively. Images of mesenteries were acquired as multiple tile scans using a HC FLUOTAR L 25×/0.95 W VISIR objective in resonant scan mode on a Leica SP8 confocal microscope. For each image, a *z*-stack was acquired at 5 μm intervals through the entire thickness of tissue containing blood and lymphatic vessels. RBCs were counted from individual stacks of images of whole mesenteries (E13; *n*=5 embryos) or along segmentally organized blood vessels covering the entire length from the mesenteric root to the intestinal wall [E13.5 (*n*=4 embryos and 5-7 vessels per embryo; total 23 vessels), E14 (*n*=7 embryos and 4-7 vessels per embryo; total 39 vessels), E16 (*n*=5 embryos and 6-9 vessels per embryo; total 37 vessels)] using the Cell Counter plugin of Fiji. Data summarized in Fig. 1D represent values for individual embryos (E13) or vessels (E13.5, E14, E16). Different categories of RBCs were assigned based on localization: intravascular (inside the blood vessels) or extravascular (outside of the blood vessels). The latter were further categorized as associated with LECs or their protrusions, or not associated. If RBCs colocalized with LEC cell bodies and/or protrusion in the same optical section(s), they were defined as associated with LECs/protrusions; otherwise they were defined as not associated.

For quantification of openings within the endothelial layer, gaps were extracted from individual single *z*-stacks from which they could be clearly observed, and their areas (*A*) were measured using the ‘Measure’ tool of Fiji. The gaps were assumed to be circular, and diameters (*d*) were calculated from measured areas 
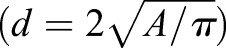
, as described previously (Hashizume et al., 2000); 294 gaps from 40 images acquired from 14 wild-type E14 mesenteries were measured.

For quantification of platelet areas, mesenteries from E14 embryos [*Clec1b^+/+^* (*n*=3) and *Clec1b^−/−^* (*n*=3)] were stained for CD41 to label platelets, and Nrp2 to label LECs and venous blood ECs. Single-tile images were acquired using a HC PL APO CS2 63×/1.30 Glyc CoRR CS3 objective and a Leica SP8 confocal microscope. For each sample, three single-tile images were taken from three different regions of the mesentery. The images were acquired at 2 μm intervals through the entire thickness of tissue containing blood and lymphatic vessels. CD41-positive areas, both total and LEC associated, were measured in maximum intensity projection images after threshold adjustment using Fiji. Optical sections to be included for each measurement (e.g. covering the thickness of a lymphatic vessels) were defined individually from each *z*-stack.

The data were calculated using Microsoft Excel for Mac 2011, and summarized and graphed using GraphPad Prism 6 for Mac.

### Flow cytometry

Mesenteries of E14 *Pdgfrb-eGFP* embryos were dissected and dissociated at 37°C and 550 rpm for 5-10 min with 2 mg/ml Collagenase IV (Life Technologies) and 0.2 mg/ml DNase I (Roche) in PBS supplemented with 0.2% fetal bovine serum (FBS; Gibco). Digests were quenched by adding 2 mM EDTA, filtered through a 70 µm nylon filter (BD Biosciences) and washed twice with FACS buffer (0.5% FBS and 2 mM EDTA in PBS). Digests were incubated for 15 min with 5 µg/ml rat anti-mouse CD16/CD32 IgG (clone 93, eBioscience) to block Fc-receptor binding and subsequently stained with rat anti-PECAM1/CD31-PE-Cy7 (0.67 µg/ml clone 390, eBioscience), hamster anti-PDPN-eF660 (2 µg/ml clone eBio8.1.1., eBioscience), rat anti-CD11b-eF450 (4 µg/ml clone M1/70, eBioscience) and rat anti-CD45-eF450 (4 µg/ml clone 30-F11, eBioscience) antibodies for 30 min on ice. Sytox Blue (1 mM; Life Technologies) was used to assess cell viability. Single stained samples were used for compensation. Samples were analyzed on a BD Cytoflex S flow cytometer equipped with CytExpert software (BD Biosciences) and processed using FlowJo 10.3 software (FlowJo, LLC).

## Supplementary Material

Supplementary information

Supplementary information
