# Supplementary material for: Transient loss of venous integrity during developmental vascular remodeling leads to red blood cell extravasation and clearance by lymphatic vessels
Source: Development. 2018 Feb 1;145(3):dev156745. doi: 10.1242/dev.156745 (PMC5818000; doi:10.1242/dev.156745)
Supplement: Supplementary information [file develop-145-156745-s1.pdf]

Supplementary figures

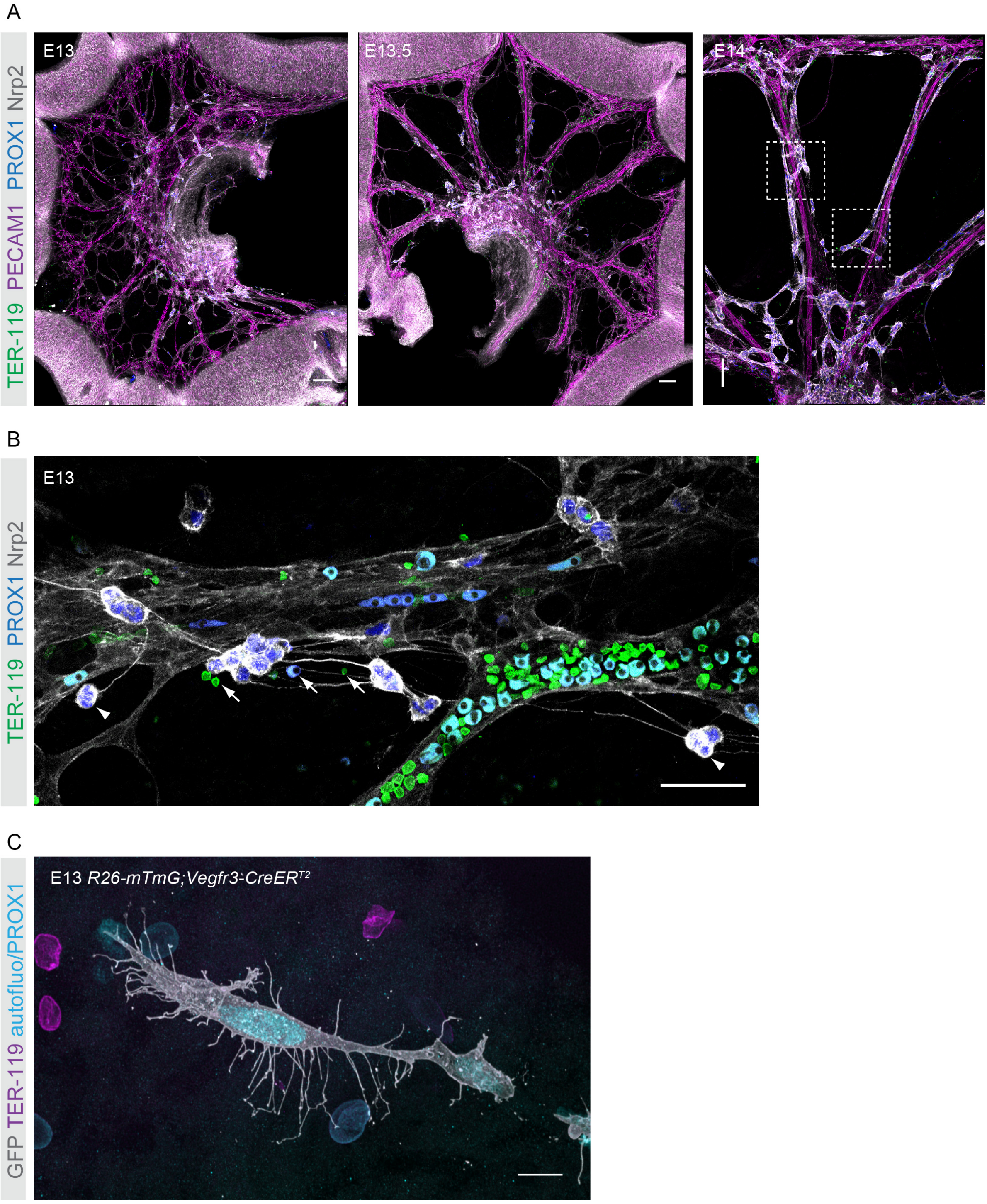

Figure S1. Analysis of extravascular RBCs in the mesentery.

(A) Representative maximum intensity projections of whole-mount stained E13, E13.5 and E14 mesenteries used for quantification of intra/extravascular RBCs. Antibodies are indicated. Boxed areas are magnified in Fig. 1C.

Development • Supplementary information

(B) Representative maximum intensity projection of whole-mount stained E13 mesentery showing rare extravascular RBCs (arrows) and their locations relative to LEC clusters. Many LEC clusters are not associated with RBCs at this stage (arrowheads). Antibodies are indicated.

(C) Maximum intensity projection of a LEC cluster in E13 mesentery extending membranous protrusions of which some are in direct contact with nucleated (TER-119^{low}) extravascular RBC. Antibodies are indicated.

Scale bars represent 100 μm (A), 50 μm (B) and 10 μm (C).

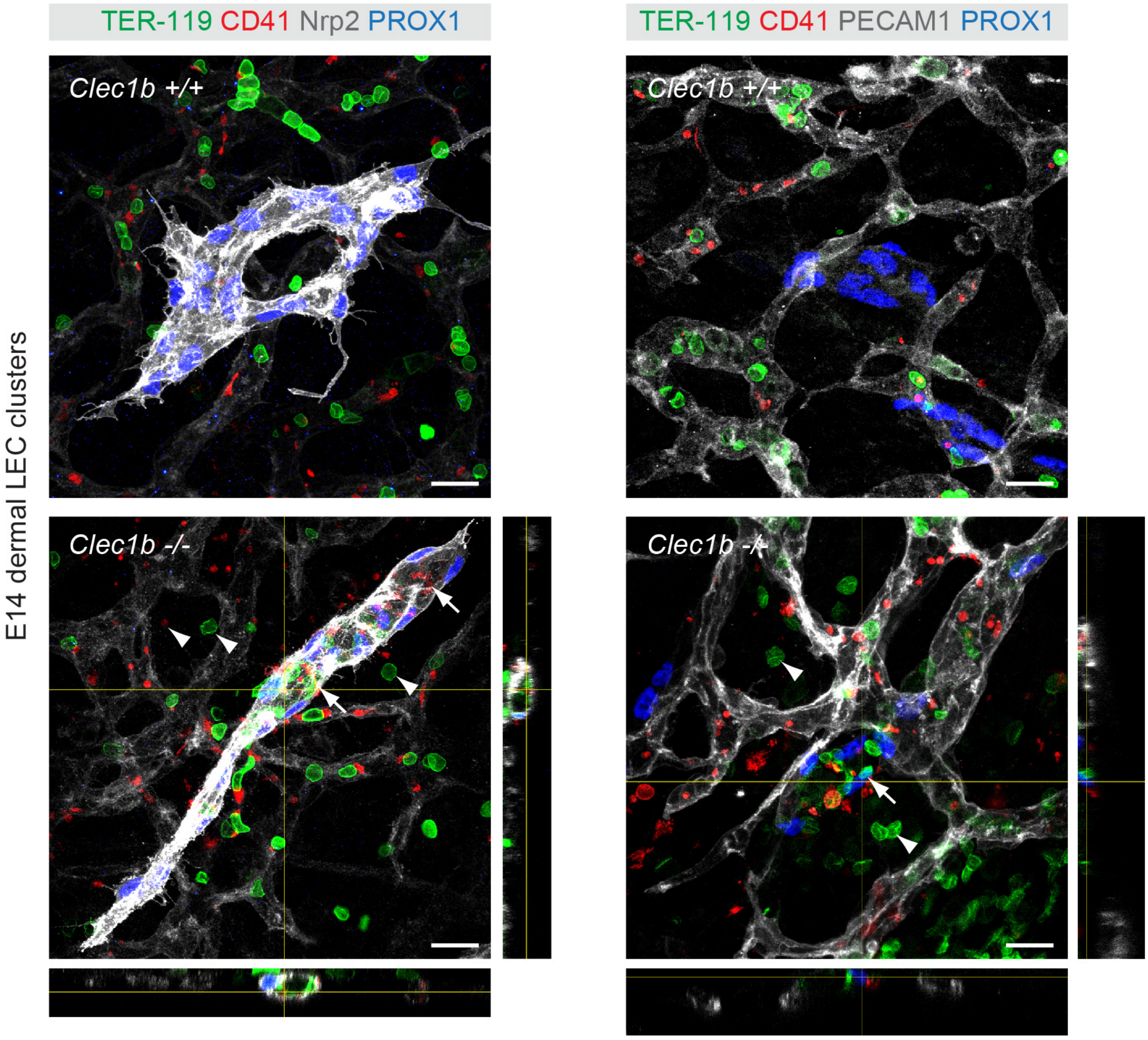

Figure S2. Association of RBCs and platelet with dermal LEC clusters in *Clec1b*$^{-/-}$ skin. Whole-mount immunofluorescence staining of E14 wild type and *Clec1b*$^{-/-}$ dorsal skins using the indicated antibodies. TER-119^{+} RBCs and CD41^{+} platelets are found outside of Nrp2^{low} (left panel) and PECAM1^{+} (right panel) blood vessels (arrowheads) and engulfed inside Nrp2^{high} LEC clusters (left panel; arrow) in *Clec1b*$^{-/-}$ but not in wild type skins. Z-views at the indicated position are shown. Scale bars represent 20 µm.

Development • Supplementary information

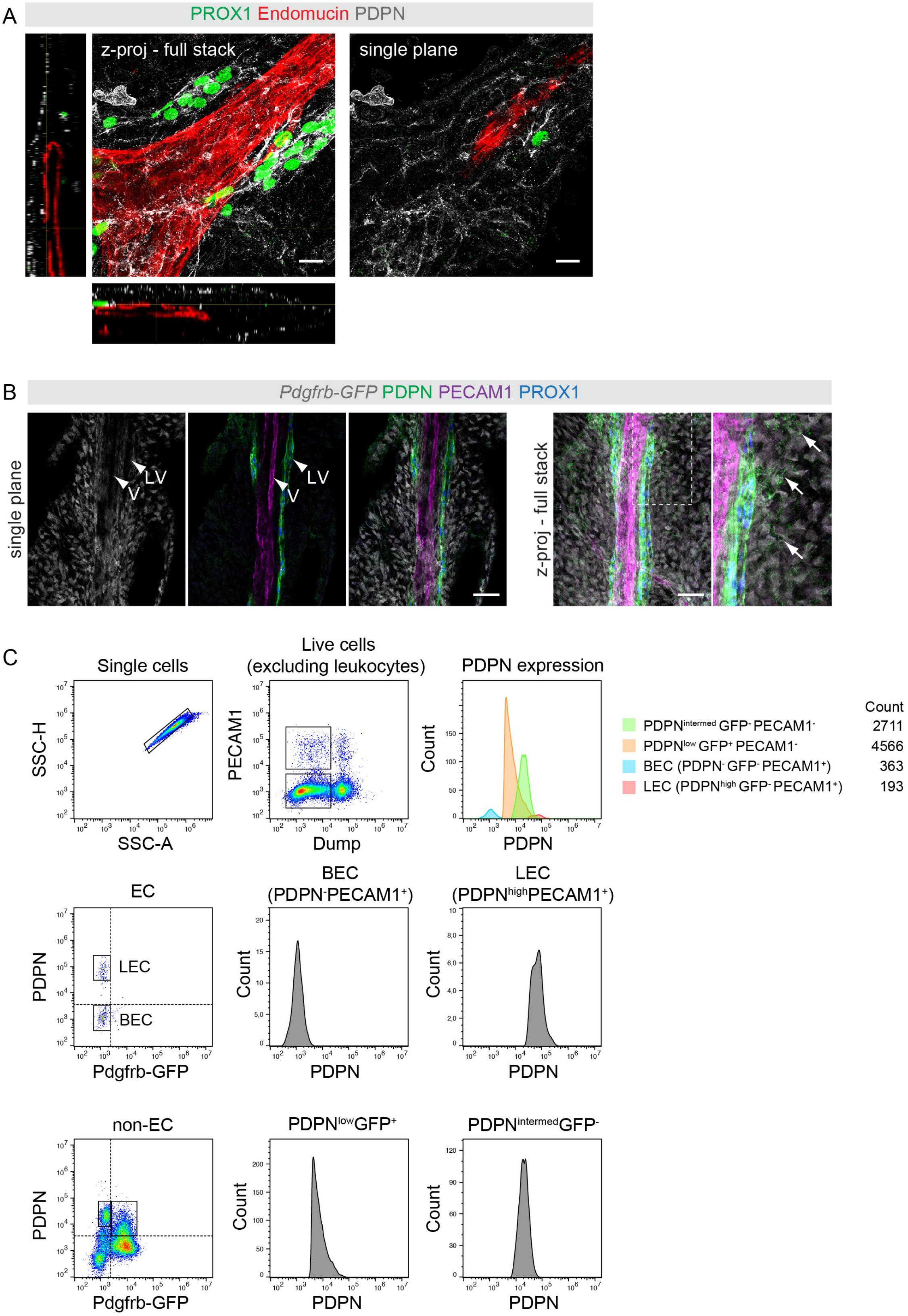

Figure S3. Podoplanin expression in the developing mesentery.

(A) Whole-mount staining of E14 mesentery for the indicated antibodies showing podoplanin^{+}PROX1^{+} LECs and podoplanin^{+}PROX1^{-} stromal cells. Z-projection of a confocal

Development • Supplementary information

stack and Z-views at the indicated positions are shown on the left. The distance between the podoplanin^{+}PROX1^{-} cell layer (shown on the right as a single plane image) and the Endomucin^{+} vein is about 12 µm.
(B) Whole mount staining of an E14 *Pdgfrb-eGFP* mesentery for the indicated antibodies showing broad expression of GFP in mural cells and mesenteric stromal cells but not in ECs (single plane, arrowhead; LV, lymphatic vessel; V, vein). The maximum intensity projection of a tiled z-stack over a distance of 32 µm reveals the presence of podoplanin expressing stromal cells (arrows). Boxed area is magnified on the right.
(C) Flow cytometric analysis of podoplanin expression in PECAM1 positive endothelial cells and PECAM1 negative stromal cells from E14 *Pdgfrb-eGFP* mesenteries. FACS plots are representative of 12 mesenteries.
Scale bars represent 20 µm (A) and 50 µm (B).

Supplementary Table S1. Image acquisition details.

Figure	Objective	Tile scan
1A-C	HC PL APO 63x/1.30 Glyc CORR CS2	No
1F	HC PL APO 63x/1.30 Glyc CORR CS2	No
1G	Plan-Apochromat 63x/1.4 Oil DIC M27	No
2A	HC PL APO 40x/1.10 W motCORR CS2	Yes (6x5, 5x6, 5x9)
2B	HC PL APO 63x/1.30 Glyc CORR CS2	No
3A-B	HC PL APO 63x/1.30 Glyc CORR CS2	No
4A	HC PL APO 63x/1.30 Glyc CORR CS2	No
4C	HC PL APO 63x/1.30 Glyc CORR CS2	No
S1A	HC FLUOTAR L 25x/0.95 W 0.17 VISIR	Yes (5x4, 6x5, 2x3)
S1B	HC PL APO 40x/1.10 W motCORR CS2	Yes (1x3)
S1C	HC PL APO 63x/1.30 Glyc CORR CS2	No
S2	HC PL APO 63x/1.30 Glyc CORR CS2	No
S3A	HC PL APO 63x/1.30 Glyc CORR CS2	No
S3B	HC PL APO 63x/1.30 Glyc CORR CS2	Yes (2x2)
Movie 1	HC PL APO 63x/1.30 Glyc CORR CS2	No
Movie 2	Plan-Apochromat 63x/1.4 Oil DIC M27	No
Movie 3	Plan-Apochromat 63x/1.4 Oil DIC M27	No
Movie 4	HC PL APO 63x/1.30 Glyc CORR CS2	No
Movie 5	HC PL APO 63x/1.30 Glyc CORR CS2	No

Development • Supplementary information

Movies

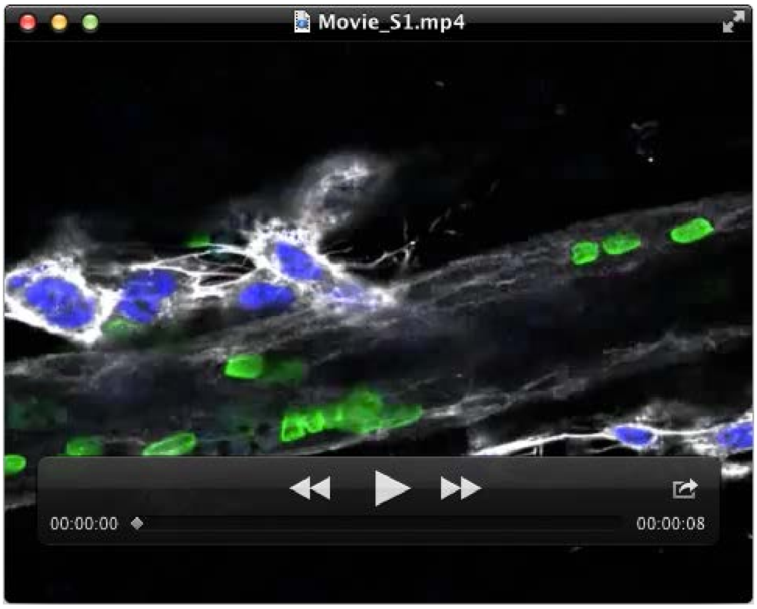

Movie 1. Z-stack of confocal images of E14 mesenteric vessels stained for markers of venous/lymphatic ECs (Nrp2) and RBCs (TER-119).

Series of confocal images of E14 mesenteric vessels stained for Nrp2, PROX1 and TER-119, showing interactions between mesenteric LECs and extravascular RBCs. The movie goes through 7.28 μm distance. Images within the series were acquired at 0.28 μm intervals and the movie is displayed at 3 frames per second.

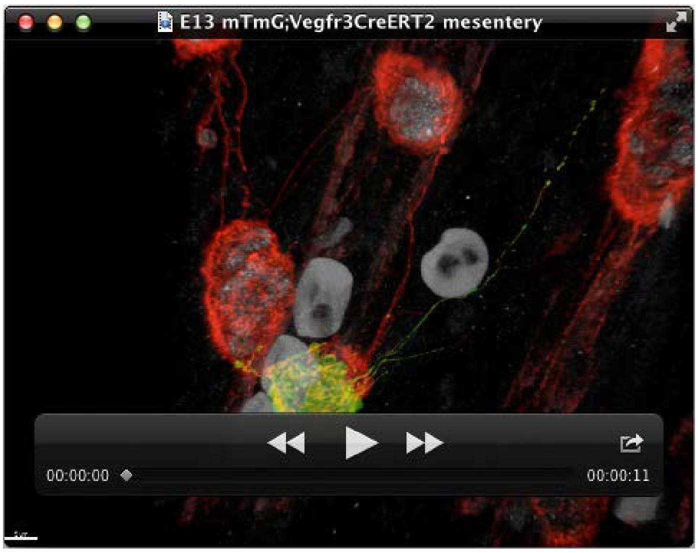

Movie 2. 3D reconstruction of E13.5 mesenteric vessels showing close association between LEC and extravascular RBC.

A movie showing 3D reconstruction of deconvolved confocal images of an E13.5 *R26-mTmG;Vegfr3-CreER*^{T2} mesentery stained for GFP (green), Nrp2 (red), PROX1 (grey; LEC nuclei) or visualized by autofluorescence (grey; RBC). The image stack was acquired at 0.20 µm intervals covering a z distance of 23.2 µm. 3D surface rendering was applied based on signal intensity to highlight close association of a LEC protrusion and RBC. A corresponding z-projection is shown in Fig. 1F. The movie is displayed at 25 frames per second.

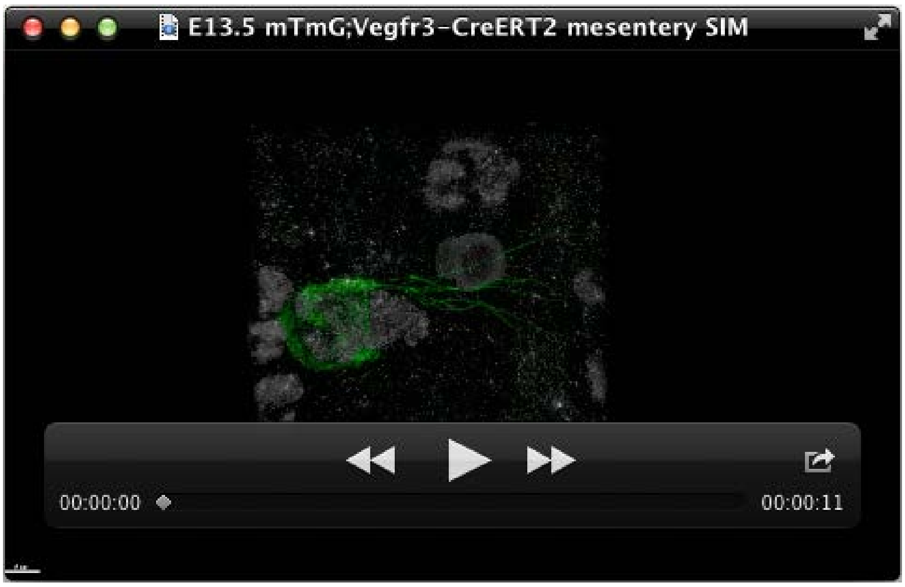

Movie 3. 3D reconstruction of structural illumination image stack showing close association between LEC cluster and extravascular RBC in an E13.5 mesentery.

A movie showing 3D reconstruction of an image stack acquired by structural illumination microscopy of whole mount E13.5 *R26-mTmG;Vegfr3-CreER*^{T2} mesentery stained for GFP (green), PROX1 (grey; LEC nuclei) or visualized by autofluorescence (grey; RBC). The image stack was acquired at 0.11 µm intervals covering a z distance of 10.1 µm. 3D surface rendering was applied based on signal intensity. A corresponding z-projection is shown in Fig. 1G. The movie is displayed at 25 frames per second.

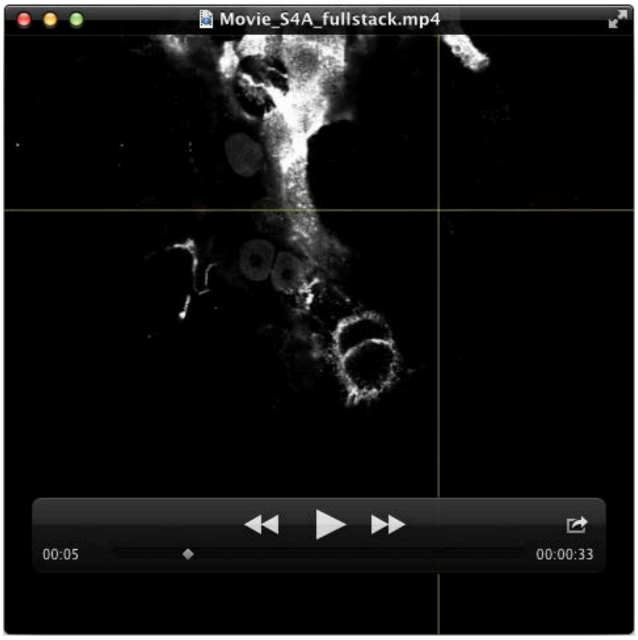

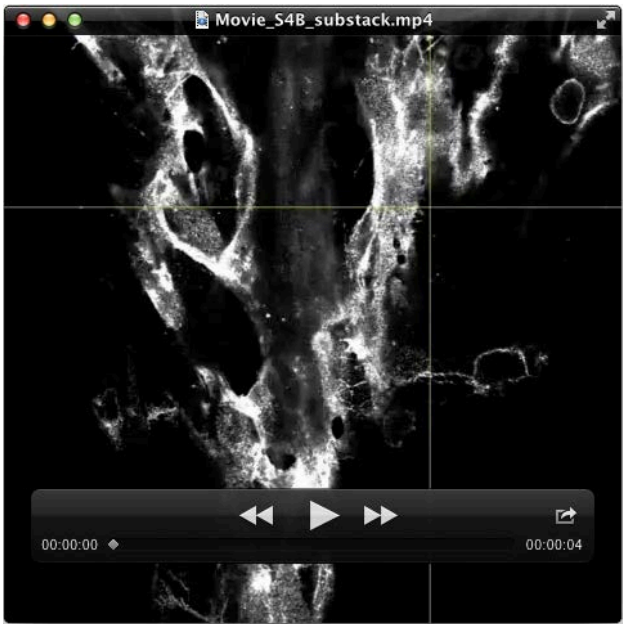

Movie 4. Z-stack of confocal images of E14 mesenteric vessels stained for the pan-EC marker PECAM1.

Series of confocal images of E14 mesenteric vessels stained for PECAM1, showing intercellular gaps in the venous endothelium. The movie goes through 28.28 μm distance covering the entire thickness of tissue containing blood vessels (Movie 4A), or 4.2 μm distance covering the venous endothelial layer at the marked position (yellow arrow) to highlight an intercellular gap (Movie 4B). Corresponding z-projections are shown in Fig. 3A (left panels). Images within the series were acquired at 0.28 μm intervals and the movie is displayed at 3 frames per second.

Development • Supplementary information

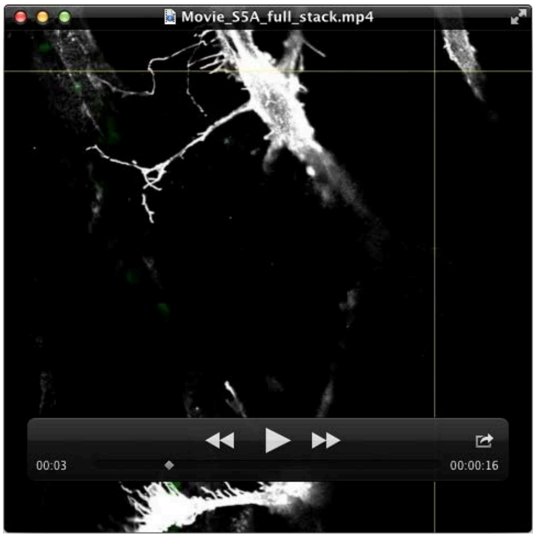

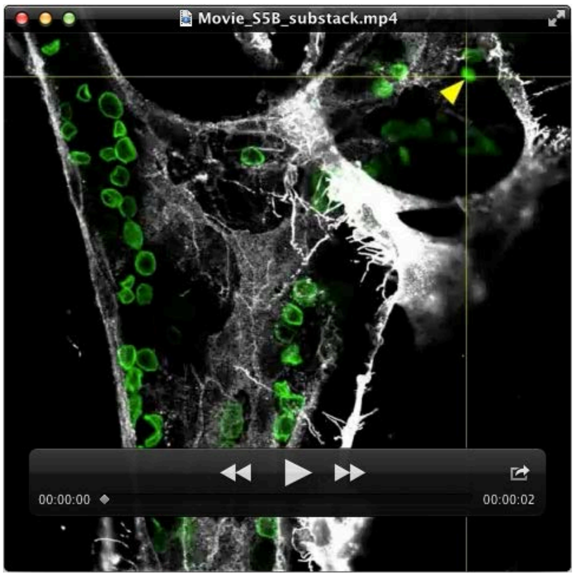

Movie 5. Z-stack of confocal images of E14 mesenteric vessels stained for markers of venous/lymphatic ECs (Nrp2) and RBCs (TER-119).

Series of confocal images of E14 mesenteric vessels stained for Nrp2 and TER-119, showing intercellular gaps in the venous endothelium. The movie goes through 13.72 μm distance covering the entire thickness of tissue containing blood and lymphatic vessels (Movie 5A), or 1.96 μm distance covering the venous endothelial layer at the marked position (yellow arrow) to highlight an intercellular gap (Movie 5B). Corresponding z-projections are shown in Fig. 3B (right panels). Images within the series were acquired at 0.28 μm intervals and the movie is displayed at 3 frames per second.

Development • Supplementary information
